# Prediction of hot spots towards drug discovery by protein sequence embedding with 1D convolutional neural network

**DOI:** 10.1371/journal.pone.0290899

**Published:** 2023-09-18

**Authors:** Youzhi Zhang, Sijie Yao, Peng Chen

**Affiliations:** 1 School of Computer and Information, Anqing Normal University, Anqing, China; 2 University Key Laboratory of Intelligent Perception and Computing of Anhui Province, Anqing Normal University, Anqing, China; 3 National Engineering Research Center for Agro-Ecological Big Data Analysis & Application, Information Materials and Intelligent Sensing Laboratory of Anhui Province, Institutes of Physical Science and Information Technology & School of Internet, Anhui University, Anhui, China; Jeonbuk Natiomal University, REPUBLIC OF KOREA

## Abstract

Protein hotspot residues are key sites that mediate protein-protein interactions. Accurate identification of these residues is essential for understanding the mechanism from protein to function and for designing drug targets. Current research has mostly focused on using machine learning methods to predict hot spots from known interface residues, which artificially extract the corresponding features of amino acid residues from sequence, structure, evolution, energy, and other information to train and test machine learning models. The process is cumbersome, time-consuming and laborious to some extent. This paper proposes a novel idea that develops a pre-trained protein sequence embedding model combined with a one-dimensional convolutional neural network, called Embed-1dCNN, to predict protein hotspot residues. In order to obtain large data samples, this work integrates and extracts data from the datasets of ASEdb, BID, SKEMPI and dbMPIKT to generate a new dataset, and adopts the SMOTE algorithm to expand positive samples to form the training set. The experimental results show that the method achieves an F1 score of 0.82 on the test set. Compared with other hot spot prediction methods, our model achieved better prediction performance.

## 1. Introduction

Protein-protein interaction is the critical link in the maintenance of cellular life activities and the exertion of cellular functions [1]. In this process, hot spot residues, which contribute most of the binding free energy, are essential for mediating protein interactions and stabilizing the structure of the protein complex [2]. Accurate identification of these hot spots is conducive to understanding the mechanism of protein-protein interactions, conducting protein engineering research, and designing drug targets [3, 4]. In recent decades, alanine mutagenesis scanning has been the most widely used biological experiment for hot spot identification [5]. However, due to its long experimental cycle and high resource consumption, computational and mathematical models are gradually being used to help us understand the omics data generated by high-throughput experimental techniques, and even increasingly used to identify hot spot residues [6].

Currently, many machine learning methods have been developed to predict hot spot residues. Wang et al. [7] extracted 600-dimensional features to represent amino acid residues, including sequence features, structural features, energetic features, and exposure features. Then, the author used mRMR and sequential forward feature selection algorithm to select a set of 26 optimal features, and finally used Extreme Gradient Boosting (XGBoost) for prediction. Hu et al. [8] extracted the physicochemical and biochemical properties of each amino acid combined with pseudo-amino acid composition and relative solvent surface area feature as molecular descriptors to describe each amino acid residue in protein. Ye et al. [9] used the RF algorithm to select the optimal 58-dimensional feature subset, which includes microenvironment and network features, and then applied the support vector machine (SVM) to construct the model. Qiao et al. [10] generated 82 features, including 10 physicochemical properties, B-factor, 36 bound features, 5 evolutionary conservation features, and 30 solvent-accessible surface features, and then built the hotspot prediction model by a hybrid feature selection strategy that includes 3 different feature selection methods: F-score, mRMR and decision tree. To construct SpotOn [11] prediction model, Moreira et al. calculated 881 features: 35 structure-based features and the other evolutionary/sequence-based features. In these experiments, feature engineering is always an important procedure and is widely studied. Researchers hope to obtain as many effective features as possible to describe each amino acid residue from sequence, structure, evolution, energy, and other information. However, the process of manually extracting features is cumbersome and limited to some extent. So far, only protein sequence information can be easily obtained, and only a small part of other information, such as structural information, has been successfully resolved.

In recent years, unsupervised learning neural network models have emerged in the field of natural language processing (NLP). Pre-trained language models can solve a variety of NLP tasks in new data sets, such as text classification and translation. As for protein, it can be regarded as the carrier of the "language of life" of organisms and as a "sentence", while the amino acid residues in protein can be regarded as "characters" or "words". At this point, protein can be viewed as an object that a natural language model can handle. By applying the model to an unlabeled protein database for training, the sequence information of proteins can be learned. This information is believed to reflect the physicochemical properties of each amino acid, and also related to structure and function to some extent [12]. Compared with manual feature extraction, the neural network-based extraction method is rather convenient and fast [13], which can learn deeper semantic and contextual information. Cui et al. [14] summarized the characterization methods that can represent protein sequences in recent years, including end-to-end embedding model, non-contextual embedding methods, transfer learning methods, and other methods for specific tasks. One-hot coding is a simple end-to-end embedding coding method, and is often used as a set of features in the prediction task [15]. The representative model of non-contextual embedding is Word2Vec, which often uses the CBOW or skip-gram approach in the training process [16]. ProtVec is a commonly used tool for protein sequence embedding, which is derived from the Word2Vec method [17]. ProtVecX is proposed by Asgari et al. which uses peptide pair encoding (PPE) subsequences to extend the ProVec model to variable length protein embedding, and performs well in three protein classification tasks [18]. Notably, these three methods can only capture the local environment while ignoring the entire sequence ordering information. In other words, they are not contextualized. The specific task model usually includes graph and extract-based feature representation.

Compared with the above embedding methods, the transfer learning-based encoding model has the fastest development and has been widely used in various protein prediction tasks. The representative model is Elmo [19], which was first used in the NLP field, compared with the word embedding proposed in 2013 [20] and the Glove model proposed in 2014 [21]. The trained Elmo model can provide the corresponding word vector according to the contextual information of a word, avoiding that the same word can get the same vector representation in different sentences. Michael Heinzinger successfully migrated the Elmo model to the field of protein sequence and performed well in a number of tasks such as secondary structure and prediction of regions with intrinsic disorder [22]. Similarly, BERT was developed by Jacob Devlin. It combines the left and right contexts of all layers, pre-trains deep representations in unlabeled text, and achieves good performance on 11 NLP tasks [23]. As an improved version of BERT, BioBERT initializes the model weights from BERT, is trained on biochemical domain corpora, and then applied to biochemical text mining tasks [24].

Inspired by these researches, this paper proposes an Embed-1dCNN model, which applies the protein embedding model combined with CNN to protein hotspot residue prediction for the first time. The method first extracts the information of protein hot spot residues from four data sets. Then, the embed4117 protein sequence embedding model trained by Bepler [25] is used to extract the characteristics of each amino acid residue in the protein sequence. To make all input data with the same dimension of size, the protein sequence is cut into fragments of the same size, and then a one-dimensional convolutional neural network (1dCNN) is used to process each sliding sequence window and perform the prediction work. The flow chart is shown in Fig 2. The experimental results of our model show that our method achieves 82% of F1 score and 89% of AUC better than the previous methods on the test set.

## 2. Materials and Methods

### 2.1. Data sets

In this work, 120 protein sequences were extracted from four data sets of ASEdb [26], BID [27], SKEMPI [28], and dbMPIKT [29], ensuring that the similarities between the protein sequences were less than 30%. Furthermore, hot spot residues were defined in the same way as in Liu’s method [30], i.e., interface residues with ΔΔG> 2 kcal/mol are considered as hot spot residues. Finally, the data set called MIX is obtained (shown in Table 1). Specifically, the data set contains 349 hot spot residues, 22244 non-hot spot residues, and the ratio of hotspots to non-hotspots is about 1:63. In order to balance the used data set, non-hotspots are resampled at the interval of 63, and 63 subsets with 349 non-hotspots are obtained. Finally, the MIX (new) dataset with the balanced hotspots and non-hotspots is obtained (as shown in Table 2). The work randomly sampled 1/5 of MIX (new) as a test set, and 1/5 of the remaining samples as a validation set. Since deep learning is more inclined to deal with the problem of large amounts of data, the interval sampling method was further used for the negative sample set to expand the negative data in the training set. At the same time, the SMOTE algorithm [31] is used to synthesize new hotspot samples. The details of the new data sets are shown in Table 3.

**Table 1:** Composition of MIX set.

Data set	Number of sequences	Hot spots	Non-hot spots	Total	Ratio (pos: neg)
MIX	120	349	22244	22593	≈ 1:63

**Table 2:** Composition of MIX (new) set.

Data set	Hot spots	Non-hot spots	Total
MIX (new)	349	349	698

**Table 3:** The final data sets used in this work.

Data set	Hot spots	Non-hot spots	Total
Training set	232+1378 (SMOTE)	214+349*4	3220
Validation set	49	63	112
Test set	68	72	140
Total	349+1378	1745 (349×5)	3472

#### 2.1.1. Smote algorithm

**Fig 1:**
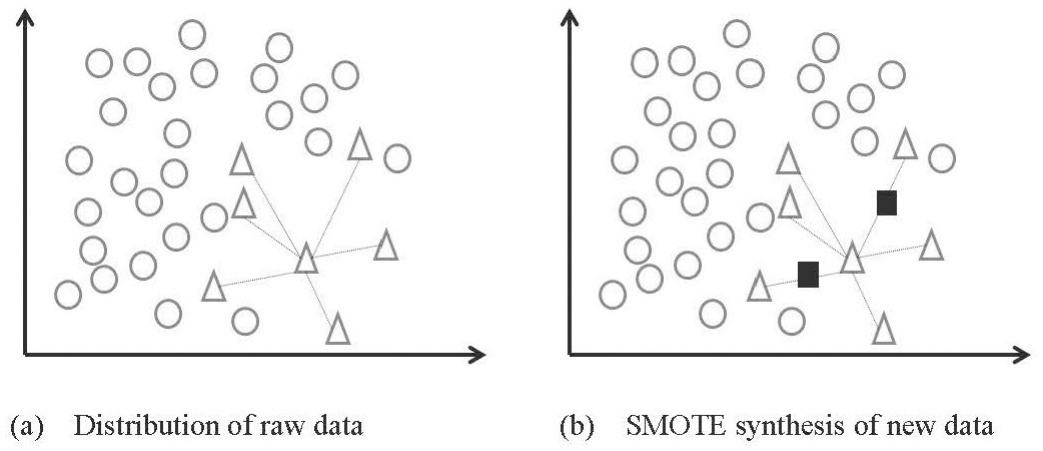
Illustrates SMOTE for generating new data. (a) shows the sample points distributed in the original space, where the circle represents majority samples, and the triangle represents minority samples. The black boxes in (b) represent the synthesized new sample points.

The SMOTE (Synthetic Minority Over-sampling Technique) algorithm was used to generate new data from existing data using established rules. Compared to direct copying of minority samples, this technique is less likely to cause overfitting. A schematic diagram of the SMOTE algorithm is shown in Fig 1.

The SMOTE algorithm is described as follows:

1. For minority samples, the SMOTE algorithm randomly selects a sample point *X*_*old*_, and calculates its distances to all minority samples based on Euclidean distance. Then set the *k* nearest-neighbor sample points of point *X*_*old*_ are set as *X*_*1*_, *X*_*2*_, *X*_*3*_,…, *X*_*n*_,…, *X*_*k*_, respectively.
2. *X*_*n*_ is randomly selected from the *k* nearest neighbors and connected to *X*_*old*_. Then, a random number *r* between (0, 1) is generated. Finally, a new minority sample is generated according to the following formula:

$$X_{new}=X_{old}+r*\left(X_{n}-X_{old}\right),\;\text{where}\;r=\text{random}\left(0,\;1\right).$$ (1)

As a result, a total of 1378 new hotspot samples have been generated by the SMOTE algorithm, based on 232 hotspot samples, so that the number of hotspots and non-hotspots in the training set is balanced.

#### 2.1.2. Interval sampling

In order to collect non-hotspots more reasonably and to make the experimental results repeatable, this work samples the non-hotspots at equal intervals. Since the number of non-hotspots is about 63 times the number of hotspots, one sample is taken every 63 negative hotspots. Using this method, a total of 1745 (349×5) non-hotspots are collected.

### 2.2. Embed-1dCNN method for protein hot spot residues prediction

**Fig 2:**
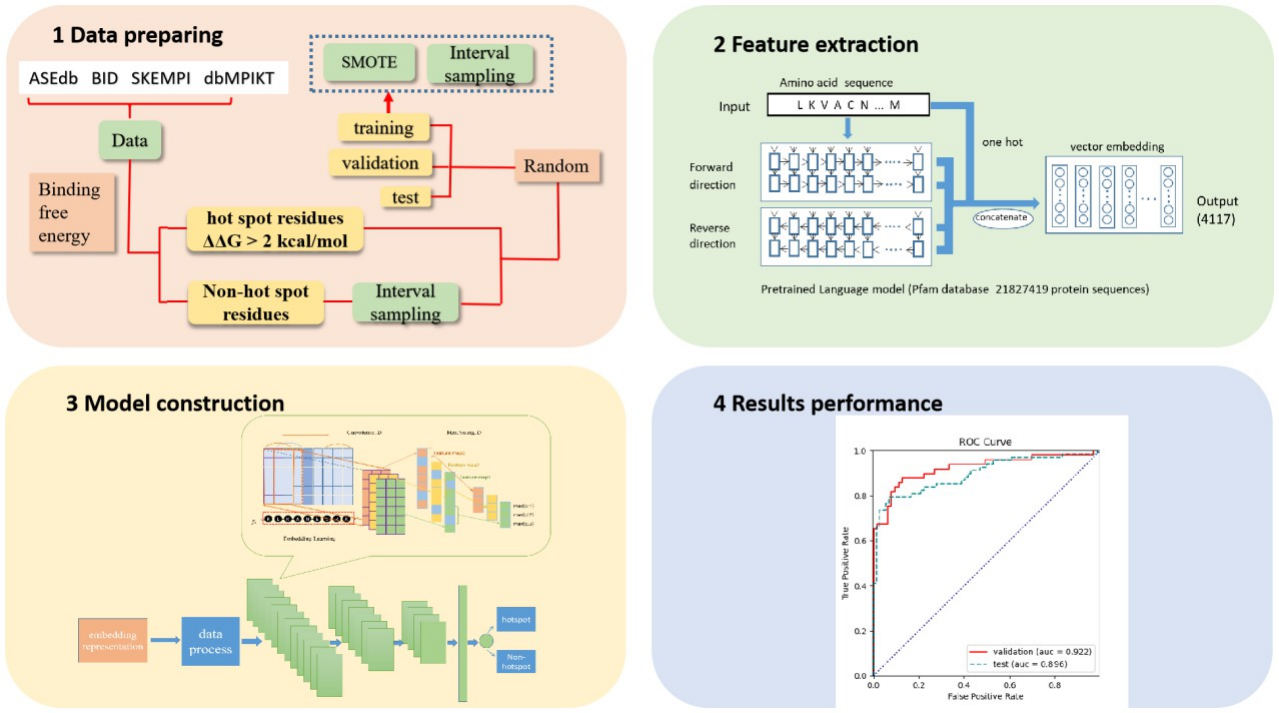
Overall framework of Embed-1dCNN. Step 1: Obtain data from the four sets of protein hotspots to generate the training, validation and test sets; Step 2: Put protein sequences into the pre-trained protein sequence embedding model to obtain amino acid descriptors; Step 3: Separate protein sequences with specific length and place sliced sequences into three layers of a one-dimensional convolutional neural network for prediction; Step 4: Evaluate the performance of the model on the validation and test sets.

This paper proposes an Embed-1dCNN model that applies one protein embedding model combined with CNN. The Embed-1dCNN workflow is shown in Fig 2.

#### 2.2.1. Protein sequence embedding model

**Fig 3:**
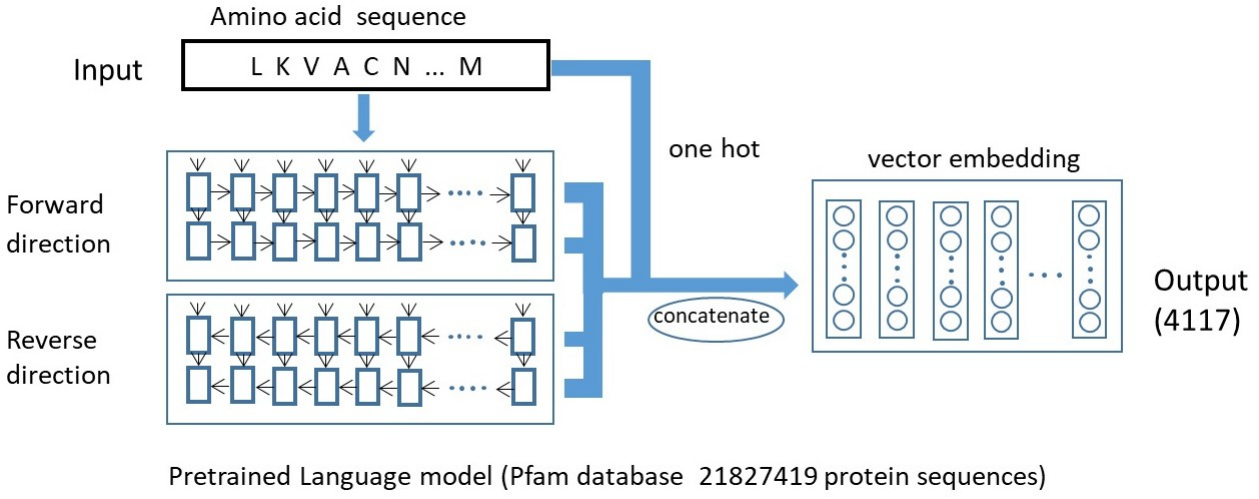
Pre-trained protein language model.

Unlike traditional feature engineering methods, this paper uses a pre-trained protein sequence embedding model (Embed4117) to obtain the feature vectors of amino acid residues in protein sequences. This model was trained by Tristan Bepler [25]. Its structure is shown in Fig 3.

Embed4117 consists of a two-layer LSTM network with 1024 LSTM units in each layer. The network incorporates forward and backward propagation processes that can capture information from protein sequences in both forward and backward directions. Embed4117 is trained on the Pfam database (Protein Family Database). There are 21,827,419 protein sequences in Pfam, which have been classified into different families using multiple sequence alignments and hidden Markov models [32]. By inputting a protein sequence into this pre-trained model, 4096-dimensional features can be obtained to describe each amino acid residue in the protein. These features are used to represent the characterization of amino acid residues. Then, each original protein sequence is expressed as a 21-dimensional one-hot vector and can be concatenated with the previous 4096 features to obtain a 4117-dimensional feature vector.

#### 2.2.2. Data processing and model construction

**Fig 4:**
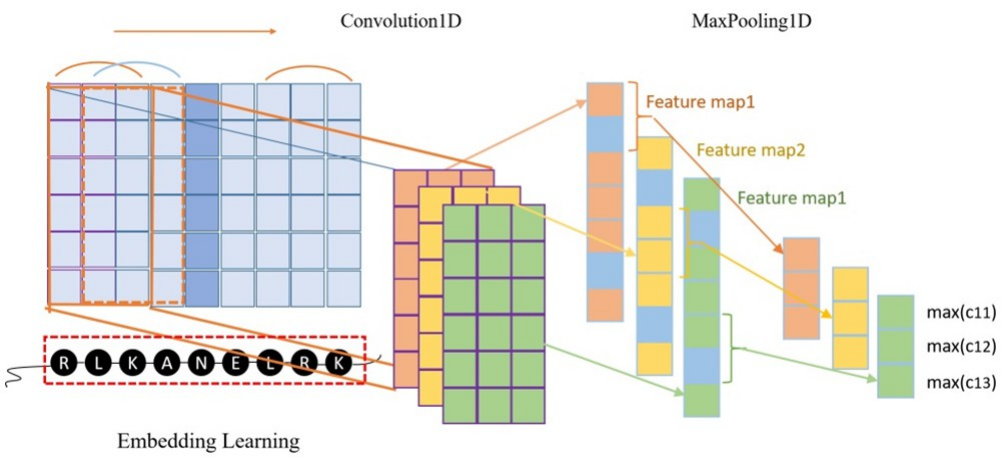
One-dimensional convolution diagram.

To obtain a feature map that represents amino acid residues in the same scale, sliding windows of different sizes (21, 23, 25…39, 41) are applied to represent the amino acid residue at the center position. As shown in Fig 4, the red box is a typical sliding protein window. It should be noted that if the number of residues to the left or right of the central amino acid residue is not enough to meet the requirements of the sliding window size, then the empty position is described as a 4117-dimensional feature vector whose element values are all 0. Unlike the two-dimensional convolution, the data of protein sequences belong to the one-dimensional sequence structure, so this work adopts the 1dCNN method [33]. The main difference between these two types of convolution is that the kernel height of 1dCNN is consistent with the feature size, whose convolution kernel only slides in the horizontal direction, but not in the vertical direction. The process of one-dimensional convolution is shown in Fig 4.

**Fig 5:**
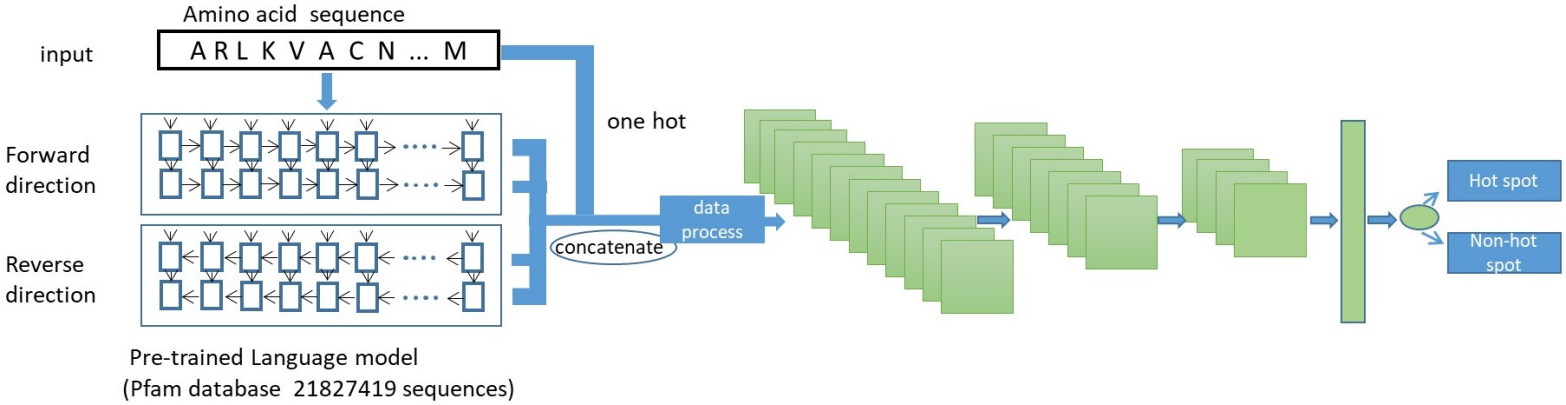
Illustration of the Embed-1dCNN model.

In this work, a total of three layers of one-dimensional convolutional operations are used, as shown in the green part of Fig 5. Among these three layers, the number of convolution kernels is 64, 16, and 4, respectively; the sizes of the convolution kernels are 7, 5, and 3, respectively; the pool used is maximum pooling with size 2; the activation function used is the ’relu’ activation function; the padding is the ’same’ padding; and the value of dropout is set to 0.5, which means that half of the connections between neurons are discarded, which can effectively avoid the occurrence of overfitting. The number of neurons in the fully connected layer is 8, and the activation function is used to generate the final probability value. The network optimizer uses the Rmsprop (Root Mean Square Prop), and the loss function is the binary cross-entropy loss. Since the protein sequence used as input data is simple, in order to avoid too much fluctuation each time, the model adopts the method of early stopping and saving itself, which makes the validation set yield the best experimental results each time. The F1 score is used as the evaluation criterion in the process. The structure of the Embed-1dCNN model is shown in Fig 5.

### 2.3. Evaluation criteria

This paper uses the commonly used machine learning evaluation criteria, including accuracy, recall, precision, F1 score, and specificity, to evaluate the performance of the model [34]. In addition, this paper draws the receiver operating characteristic (ROC) curve and calculates the corresponding AUC value [35]. The detailed calculations are:

$$\text{Acc}=\frac{\text{TP}+\text{TN}}{\text{TP}+\text{FP}+\text{TN}+\text{FN}}$$ (2)

$$\text{Recall}=\frac{\text{TP}}{\text{TP}+\text{FN}}$$ (3)

$$\text{Pre}=\frac{\text{TP}}{\text{TP}+\text{FP}}$$ (4)

$$\text{Spe}=\frac{\text{FP}}{\text{FP}+\text{TN}}$$ (5)

$$\text{F}1=\frac{2\times \text{Pre}\times \text{Recall}}{\text{Pre}+\text{Recall}}$$ (6)

where TP, TN, FP, FN represent the number of true positives (correctly predicted hot spot residues), true negatives (correctly predicted non-hot spot residues), false positives (non-hot spot residues falsely predicted as hot spot residues), and false negatives (hot spot residues falsely predicted as non-hot spot residues), respectively. The ROC curve refers to the recall (sensitivity) and specificity. The AUC is the area under the ROC curve.

## 3. Results and discussion

### 3.1. Setting of hyper-parameters for the proposed method

In order to obtain the best performance of the proposed method, different combinations of hyperparameters have been investigated in this work. Tables 4–6 show the performance comparison of different hyperparameters. As shown in Table 4, it can be seen that numbers of 64, 16, and 4 achieve relatively good results with fewer parameters, therefore a set of hyper-parameters, (64, 16, 4), was selected in this work. Similarly, for the three layers of the method, the sizes of the convolution kernels are set to (7, 5, 3) (see Table 5), and "relu" is set as the activation function of the method (see Table 6). The performance comparison is also shown in S4 File.

**Table 4:** Prediction performance of Embed-1dCNN on the validation set and test set.

Data sets	Acc	Recall	Pre	F1	Spe
validation	0.8929	0.8289	0.9378	0.8692	0.9397
test	0.8514	0.7206	0.9662	0.8249	0.9750

**Table 5:** Performance comparison of the model with two techniques when sliding window length is 33.

Model	Acc	Recall	Pre	F1	Spe
Embed-1dCNN (SMOTE)	0.8929	0.8289	0.9378	0.8692	0.9397
Embed-1dCNN (class_weight)	0.8888	0.8364	0.9091	0.8594	0.9312

**Table 6:** Performance comparison of different classifiers on validation set.

Classifier Model	Acc	Recall	Pre	F1	Spe
KNN	0.6714	0.6471	0.6667	0.6567	0.6944
ADB	0.6643	0.6471	0.6567	0.6519	0.6806
SVM	0.7143	0.6618	0.7258	0.6923	0.7639
GBDT	0.7071	0.7059	0.6957	0.7007	0.7083
ExtraTrees	0.7214	0.7206	0.7101	0.7153	0.7222
MLP	0.7214	0.7206	0.7206	0.7206	0.7361
LR	0.7571	0.7059	0.7742	0.7385	0.8056
Embed-1dCNN (SMOTE)	0.8929	0.8289	0.9378	0.8692	0.9397

### 3.2. Experimental performance of the model Embed-1dCNN on the validation set and test set

**Fig 6:**
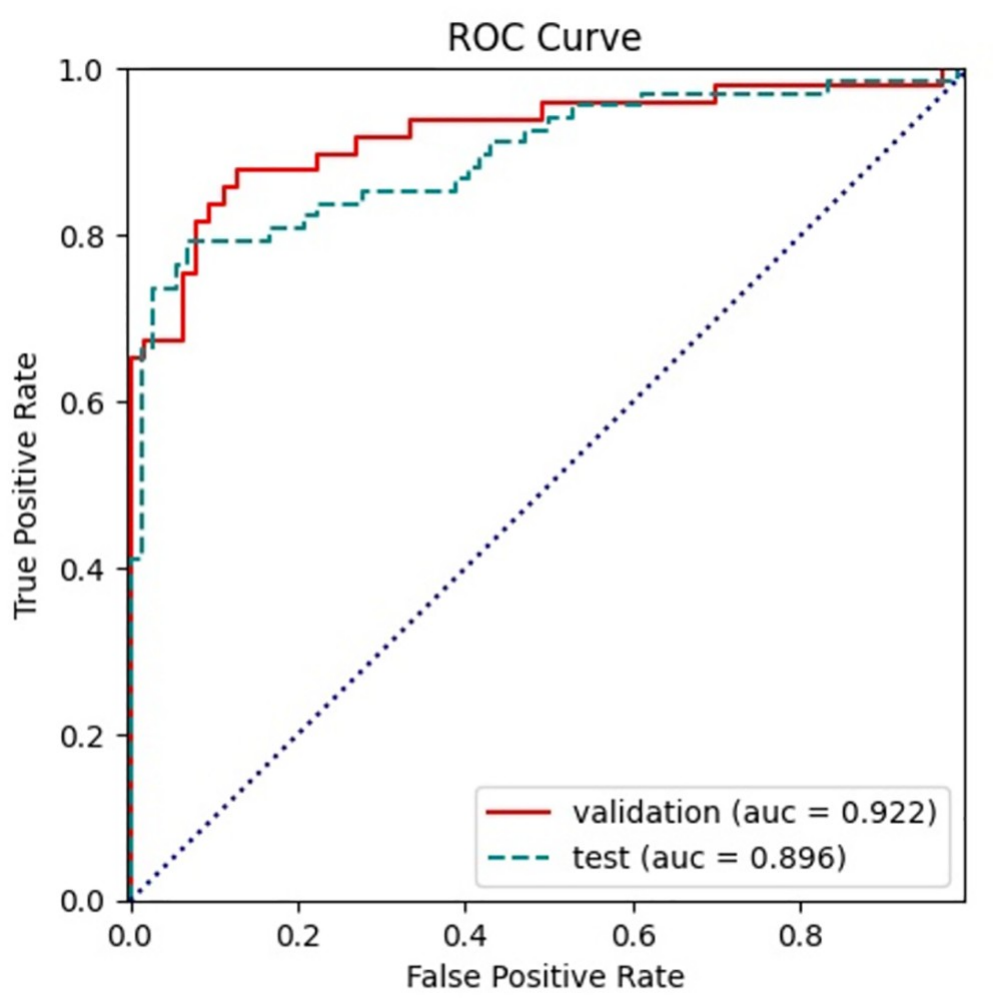
ROC curve and AUC value of validation and test set.

The experimental results on the validation set and the test set are shown in Table 4. As a comprehensive evaluation criterion, it can be seen that the F1 score on the validation set reaches 0.86, while on the test set it is 0.82. The accuracies on the validation set and the test set are 0.89 and 0.85, respectively. Note that the sliding window is 33 at this point. To observe the experimental results more intuitively, the ROC curve was drawn and the corresponding AUC value was calculated. As shown in Fig 6, the AUC value obtained on the validation set is 0.92, while on the test set it is 0.89. In general, the model has achieved relatively good experimental performance.

### 3.3. Performance influence of the number of convolutional layers

**Fig 7:**
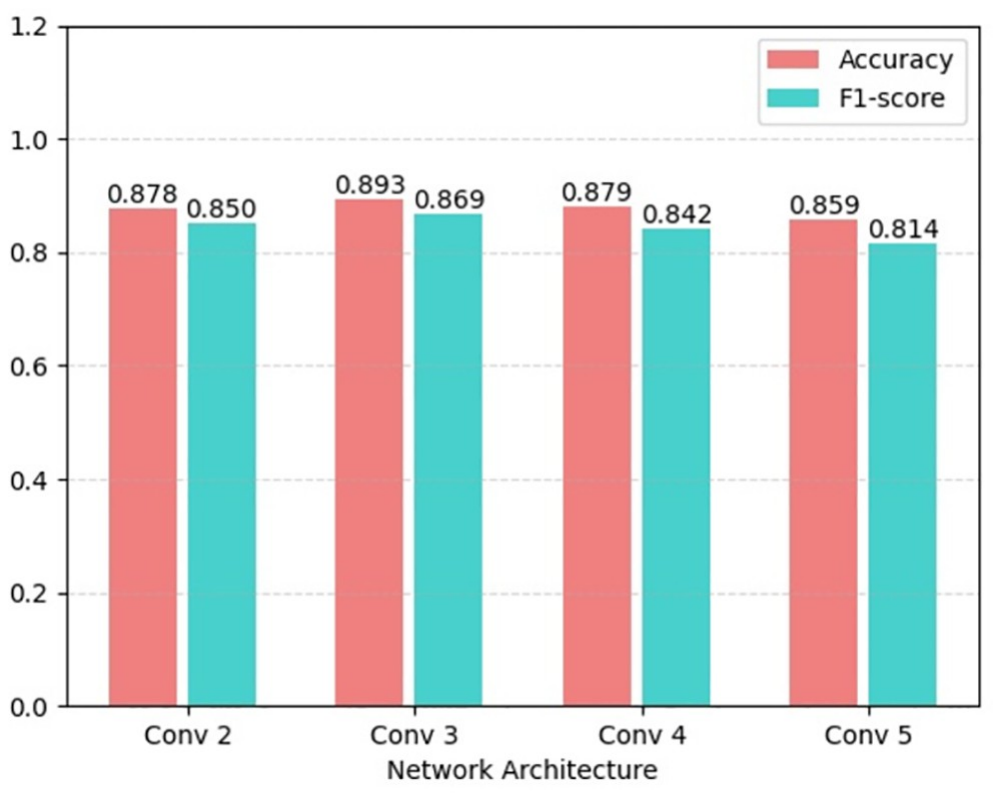
Performance comparison of the model with different convolution layers.

To explore the influence of the number of convolutional layers on the experimental results, different layers of convolutional networks were examined and performance changes were observed. In general, the more complex the structure and the more layers of the network, the easier it was to capture the deeper representations and the better the prediction performance of the network. However, this can increase the computational complexity of the network. In this work, the effect of different network layers (2, 3, 4, 5) on the performance was investigated (as shown in Fig 7). It can be seen that when the two-layer 1dCNN is increased to three layers, the model obtains a slight improvement in terms of accuracy and F1 score, but as the number of network layers is further increased, the experimental performance has decreased. When the network is 4 and 5 layers, the F1 score of the model is reduced by 2.7% and 5.5%, respectively, compared to the model with 3 layers. This may be due to the small and relatively simple data, so the complex network structure has a negative effect after increasing the number of parameters. It should be noted that the hyperparameters of the increased or decreased convolutional layer of the model are the same as those of the second layer in the original model architecture.

### 3.4 Performance comparison of SMOTE vs. ‘class_weight’ for processing imbalanced data sets under different sliding windows

**Fig 8:**
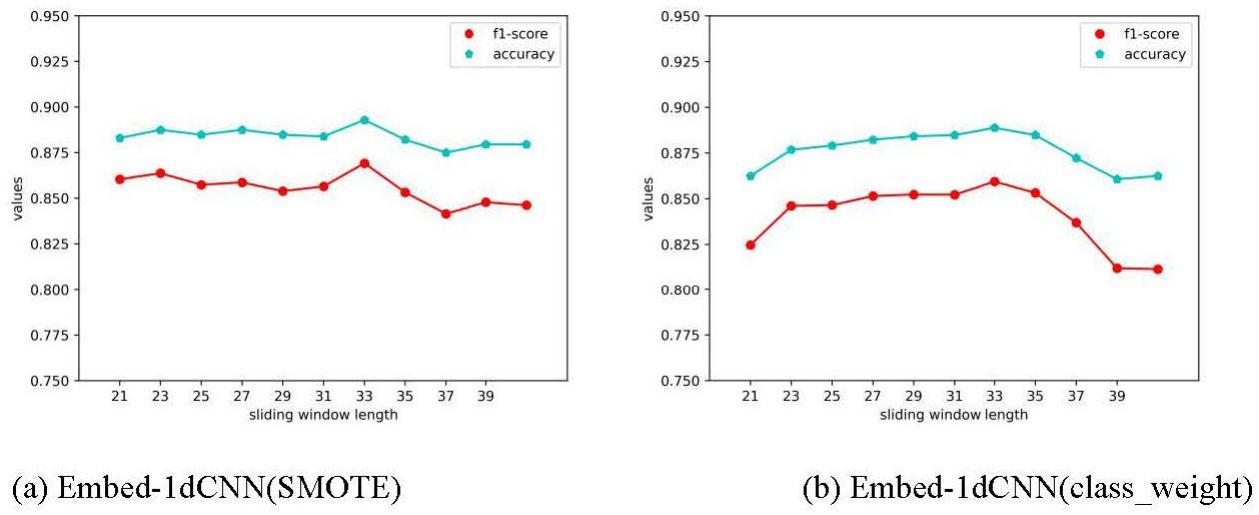
Illustration of the F1 score and accuracy of two experimental techniques under different sliding windows. (a) indicates that new samples are synthesized by the SMOTE algorithm; (b) indicates that "class_weight" is set for the model.

Since the ratio of hotspots to non-hot spots in the training set is very unbalanced, expecting to use the SMOTE techniques to synthesize hotspot samples, another technique, which sets the class weight, parameter of ’class_weight’, of samples for the neural network model to increase the cost of misclassification of minority samples, was also investigated. As shown in Fig 8, in this work, these two techniques were tried with different sliding window lengths, and the evaluation indicators include ’F1 score’ and ’accuracy’.

It can be seen intuitively that the model with both techniques gives the best performance with a sliding window length of 33. To better compare the experimental performance of these two methods with the sliding window width of 33, the detailed performance comparison can be seen in Table 5. The details of the prediction results of the two groups of models on the four evaluation indicators can be seen in S1 File.

In general, whether the SMOTE algorithm is used to balance the hotspots and non-hot spots in the training set, or a higher misclassification weight is set for the minority samples, the model has achieved good prediction performance. At the same time, the result of Embed-1dCNN (SMOTE) is better than Embed-1dCNN (class_weight) in terms of accuracy, precision, f1 score and specificity. And the recall is slightly worse than Embed-1dCNN (class_weight).

### 3.5. Performance comparison with other classifiers

To illustrate the advantages of convolutional neural networks as predictive classifiers in this work, this paper also compared with a variety of traditional machine learning algorithms on the validation set, as shown in Table 6. Compared with the 7 groups of machine learning algorithms, KNN, ADB, SVM, GBDT, ExtraTrees, MLP and LR implemented by WEKA software [36], the proposed convolutional neural network achieves an improvement in F1 score of 21.25%, 21.73%, 17.69%, 16.85%, 15.39%, 14.86% and 13.07%, respectively. In addition, MLP and LR also achieve relatively good results.

### 3.6. Performance comparison of the model before and after the expansion of the training set

To illustrate the necessity of training data expansion, the performance comparison of the model before and after training data expansion was examined. As shown in Table 7, after the training data expansion, the accuracy and F1 score of the model increased by 12.5% and 10.52%, respectively, compared to the model before the training data expansion. The improvement in prediction performance after the expansion is obvious, which indirectly shows that the convolutional neural network can learn more effective information from the training samples.

**Table 7:** Performance comparison of the model before and after training data expansion.

Model	Acc	Recall	Pre	F1	Spe
Embed4117_1dCNN	0.7679	0.8750	0.6981	0.7640	0.6734
Embed4117_1dCNN (data expansion)	0.8929	0.8289	0.9378	0.8692	0.9397

### 3.7. Compare with the state-of-the-art method

In the previous hot spot residue prediction experiments, Liu’s model achieved higher prediction performance on the BID test set. The method developed an ensemble learning method based on KNN and SVM classifiers, and conducted experiments based entirely on sequence features, which is the same type of feature used in this work. The performance comparison is shown in Table 8. It can be seen that the F1 score and accuracy of our model are better than those of Liu’s method [37]. It shows that our model has certain advantages in dealing with such problems.

**Table 8:** Performance comparison of different methods on the test set.

Method	Acc	Recall	Pre	F1	Spe
Liu’s	0.6500	0.8714	0.6040	0.7135	0.4286
Our proposed	0.8514	0.7206	0.9662	0.8249	0.9750

### 3.8. Comparison of experimental results of different protein embedding models

**Fig 9:**
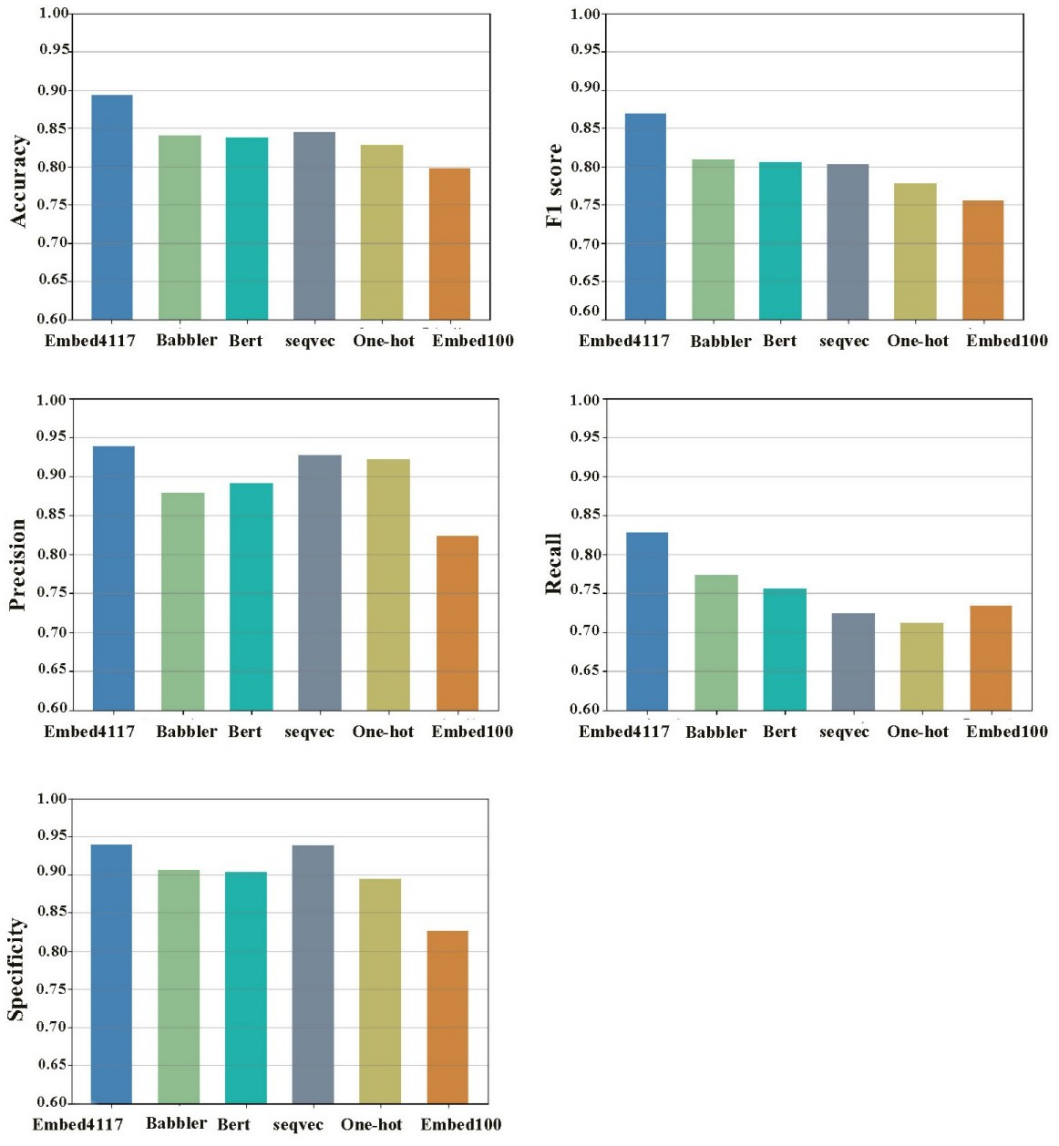
Prediction performance of different embedding models.

Furthermore, different protein sequence embedding models were investigated and compared with the Embed4117 embedding model used in our proposed method, as shown in Fig 9. The first one is called "Embed100" in the work of Tristan Bepler and Bonnie Berger [25], which also adds an encoder structure to the Embed4117. When it outputs a 4117-dimensional feature vector, it is embedded into this encoder, which contains a 3-layer BiLSTM structure and a linear layer, and outputs 100-dimensional features. The second is called "seqvec", which applied the NLP model ELMo to solve protein sequence-related problems through transfer learning and trained the protein embedding model. Third, Babbler-1900 embedding model was trained a 1900 hidden unit multiplicative long-short term memory (mLSTM) recurrent neural networks (RNNs) of amino acid character in UniRef50 [38]. The pretrained model of Babbler-1900 from TAPE was used in this work [39]. One-hot coding scheme is a common coding method in the process of data preprocessing. The processed data are usually sparsely distributed in space. In this work, amino acids in a protein are represented in the form of 21 dimensions (the 21st dimension represents all other types of amino acid residues except the common 20 amino acids). The Bert method has also been implemented in this work, called "Bert_base". The detailed description of the five embedding models can be found in S3 File.

Fig 9 shows the performance comparison of the six embedding models in terms of accuracy, F1 score, precision, recall and specificity on the validation set. It can be seen that the performance of the proposed model with embed4117 (acc = 0.89, f1 = 0.86, pre = 0.93, recall = 0.82, spe = 0.93) is better than the other five embedding models on the five indicators. Furthermore, the Babbler model performs well in F1 score and recall, while ’seqvec’ performs well in accuracy, precision and specificity. In general, Embed4117 achieves the best performance for predicting protein hotspot residues among the six embedding models. The details of the prediction results of the six groups of protein sequence embedding models can be found in S2 File.

### 3.9. Case studies

**Fig 10:**
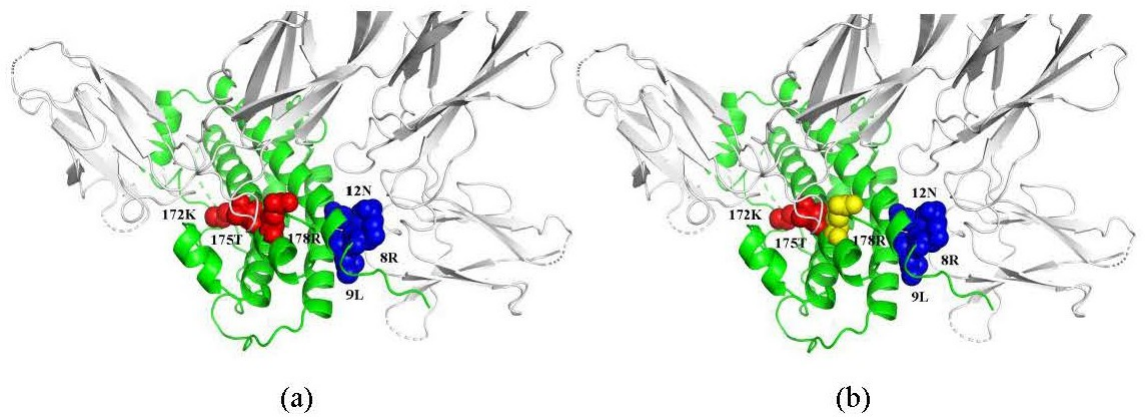
Structure visualization of PDB ID: 3HHR (chain A, B and C). (a) True hotspots and non-hotspots verified by experiment in chain A; (b) The prediction results of our method. By the proposed method, two (172K, 175T) out of three hotspots are correctly predicted, while hotspot 178R in yellow is incorrectly predicted as a non-hotspot. Three non-hotspots are all correctly predicted.

To present the prediction performance of the model in a more intuitive way, the PyMOL software was used to visualize the prediction results of proteins with PDB IDs: 3HHR and 1CZ8. As shown in Fig 10, protein 3HHR consists of three chains: chain A in green is human growth hormone (hGH), which is required for human growth and development, and chains B and C in white are the extracellular domain of human growth hormone receptors (hGHbp). In chain A there are 3 hot spots in red (172K, 175T and 178R) and 3 non-hotspots in blue (8R, 9L and 12N).

**Fig 11:**
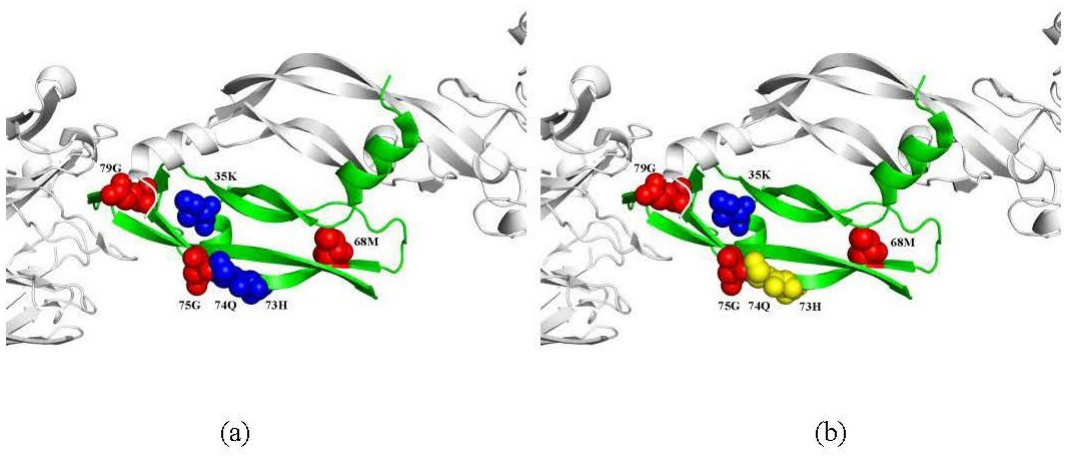
Structure visualization of PDB ID: 1CZ8 (chains V, W, L, X, H and Y). (a) True hotspots and non-hotspots verified by experiment in chain W; (b) The prediction results of our method. By the proposed method, three hotspots (68M, 75G, 79G) are correctly predicted. And the non-hotspot 35K is correctly predicted, while two non-hotspots (73H and 74Q) in yellow are incorrectly predicted as hotspots.

As shown in Fig 11, protein 1CZ8 consists of six chains: chains V and W are vascular endothelial growth factor A, chains L and X are the light chain of the neutralizing antibody, chains H and Y are the heavy chain of the neutralizing antibody. In chain W, which is shown in green (the other five chains are all shown in white), there are 3 hot spots in red (68M, 75G, and 79G) and 3 non-hot spots in blue (35K, 73H, and 74Q).

## 4. Conclusion

In this work, the protein sequence embedding learning model was applied in the field of hot spot residue prediction for the first time, and obtained good prediction performance. Specifically, the work first collect the MIX data set, and under sample the negative samples to construct training, validation and test data sets. At the same time, the SMOTE algorithm is used to generate new positive samples, which expands the training data and balances the number of positive and negative samples. Then, the protein sequence embedding model is used to extract the characterization of each amino acid residue. The protein sequence is separated and fed into a three-layer one-dimensional convolutional neural network for prediction. Compared with the previous method that predicts hot spot residues from interface residues, our model achieves good prediction performance. In addition, this work also investigates different machine learning algorithms and different sequence embedding models on experimental results. Although Embed-1dCNN has achieved better prediction performance, in actual situations, hot spot residues account for only 1%~2% of the whole protein sequence, so predicting hot spot residues from the whole sequence is the future work we expect to do.

## Supporting information

S1 File Shows the prediction results of the two groups of models on the four evaluation indicators. (DOCX)

S2 File Shows the prediction results of six groups of protein sequence embedding models. (DOCX)

S3 File Describes the five embedding models. (DOCX)

S4 File Shows the performance comparison of different hyperparameters for the proposed method. (DOCX)

S5 File (ZIP)

## References

[1] Chothia C, Janin J. Principles of protein-protein recognition. Nature. 1975 Aug 28;256(5520):705–8. doi: 10.1038/256705a0 .. 1153006

[2] Clackson T, Wells J A. A hot spot of binding energy in a hormone-receptor interface. Science, 1995, 267(5196):383–6. doi: 10.1126/science.7529940 7529940

[3] Irina S, Moreira, et al. Hot spots-A review of the protein-protein interface determinant amino-acid residues. Proteins: Structure, Function, and Bioinformatics, 2007, 68(4):803–812. 10.1002/prot.21396 17546660

[4] DeLano Warren L. Unraveling hot spots in binding interfaces: progress and challenges. curr opin struct biol, 2002, 12(1):14–20. doi: 10.1016/s0959-440x(02)00283-x 11839484

[5] Wells J A. Systematic mutational analyses of protein-protein interfaces. Methods in Enzymology, 1991, 202:390–411. doi: 10.1016/0076-6879(91)02020-a 1723781

[6] Ji Zhiwei, Yan Ke, Li Wenyang, Hu Haigen, and Zhu Xiaoliang, Mathematical and Computational Modeling in Complex Biological Systems. BioMed Research International 2017, 5958321 doi: 10.1155/2017/5958321 28386558 PMC5366773

[7] Wang H, Liu C, Deng L. Enhanced Prediction of Hot Spots at Protein-Protein Interfaces Using Extreme Gradient Boosting. Scientific Reports, 2018, 8(1). doi: 10.1038/s41598-018-32511-1 30250210 PMC6155324

[8] Hu SS., Chen P., Wang B. et al. Protein binding hot spots prediction from sequence only by a new ensemble learning method. Amino Acids 49, 1773–1785 (2017). doi: 10.1007/s00726-017-2474-6 28766075

[9] Ye L, Kuang Q, Jiang L, et al. Prediction of hot spots residues in protein–protein interface using network feature and microenvironment feature. Chemometrics & Intelligent Laboratory Systems, 2014, 131(Complete):16–21.

[10] Qiao Y, Xiong Y, Gao H, et al. Protein-protein interface hot spots prediction based on a hybrid feature selection strategy. BMC bioinformatics, 2018, 19(1): 1–16. 29334889 10.1186/s12859-018-2009-5 PMC5769548

[11] Moreira I S, Koukos P I, Melo R, et al. SpotOn: high accuracy identification of protein-protein interface hot-spots. Scientific reports, 2017, 7(1): 1–11. 28808256 10.1038/s41598-017-08321-2 PMC5556074

[12] Kulmanov M, Khan M A, Hoehndorf R. DeepGO: Predicting protein functions from sequence and interactions using a deep ontology-aware classifier. Bioinformatics, 2017, 34(4):660–668. 10.1093/bioinformatics/btx624 PMC5860606 29028931

[13] Hu Haigen, Guan Qiu, Chen Shengyong, Ji Zhiwei, Lin Yao, Detection and Recognition for Life State of Cell Cancer Using Two-Stage Cascade CNNs. IEEE/ACM Trans Comput Biol Bioinform 2020, 17(3):887–898. doi: 10.1109/TCBB.2017.2780842 29990223

[14] Cui F, Zhang Z, Zou Q. Sequence representation approaches for sequence-based protein prediction tasks that use deep learning. Briefings in Functional Genomics, 2021, 20(1): 61–73. doi: 10.1093/bfgp/elaa030 33527980

[15] José, Juan, Almagro, et al. DeepLoc: prediction of protein subcellular localization using deep learning. Bioinformatics. 2017 Nov 1;33(21):3387–3395. doi: 10.1093/bioinformatics/btx431 Erratum in: Bioinformatics. 2017 Sep 19;: . 29036616

[16] Mikolov T, Sutskever I, Chen K, et al. Distributed representations of words and phrases and their compositionality. In: Advances in neural information processing systems, Curran Associates Inc., 57 Morehouse LaneRed Hook, NY, United States. 2013, 3111–9

[17] Asgari E, Mofrad M. ProtVec: A Continuous Distributed Representation of Biological Sequences. Computer Science, 2015, 10(11):e0141287. 10.1371/journal.pone.0141287 PMC4640716 26555596

[18] Asgari E, McHardy AC, Mofrad MRK. Probabilistic variable-length segmentation of protein sequences for discriminative motif discovery (DiMotif) and sequence embedding (ProtVecX). Sci Rep. 2019 Mar 5;9(1):3577. doi: 10.1038/s41598-019-38746-w . 30837494 PMC6401088

[19] Peters M, Neumann M, Iyyer M, et al. Deep Contextualized Word Representations. In Proceedings of the 2018 Conference of the North American Chapter of the Association for Computational Linguistics: Human Language Technologies, Volume 1 (Long Papers), pages 2227–2237, New Orleans, Louisiana.

[20] Mikolov T, Chen K, Corrado G, et al. Efficient Estimation of Word Representations in Vector Space, 2013.—cite arxiv:1301.3781

[21] Pennington J, Socher R, Manning C. GloVe: Global Vectors for Word Representation. In Proceedings of the 2014 Conference on Empirical Methods in Natural Language Processing (EMNLP), pages 1532–1543, Doha, Qatar.

[22] Heinzinger M, Elnaggar A, Wang Y, et al. Modeling aspects of the language of life through transfer-learning protein sequences. BMC Bioinformatics 2019;20:723. doi: 10.1186/s12859-019-3220-8 31847804 PMC6918593

[23] Devlin J, Chang M W, Lee K, et al. BERT: Pre-training of Deep Bidirectional Transformers for Language Understanding. In Proceedings of the 2019 Conference of the North American Chapter of the Association for Computational Linguistics: Human Language Technologies, Volume 1 (Long and Short Papers), pages 4171–4186, Minneapolis, Minnesota.

[24] Lee J, Yoon W, Kim S, et al. BioBERT: a pre-trained biomedical language representation model for biomedical text mining. Bioinformatics 2020;36:1234–40. doi: 10.1093/bioinformatics/btz682 31501885 PMC7703786

[25] Bepler T, Berger B. Learning protein sequence embeddings using information from structure. 2019 arXiv:1902.08661. PMC12947628 41768824

[26] Thorn KS, Bogan AA. ASEdb: a database of alanine mutations and their effects on the free energy of binding in protein interactions. Bioinformatics. 2001 Mar;17(3):284–5. doi: 10.1093/bioinformatics/17.3.284 . 11294795

[27] Fischer TB, Arunachalam KV, Bailey D, Mangual V, Bakhru S, Russo R, et al. The binding interface database (BID): a compilation of amino acid hot spots in protein interfaces. Bioinformatics. 2003 Jul 22;19(11):1453–4. doi: 10.1093/bioinformatics/btg163 . 12874065

[28] Moal Iain H. and Fernández-Recio Juan*. SKEMPI: a Structural Kinetic and Energetic database of Mutant Protein Interactions and its use in empirical models. Bioinformatics, 2012, 28(20):p.2600–2607. doi: 10.1093/bioinformatics/bts489 22859501

[29] Liu Q, Chen P, Wang B, Zhang J, Li J. dbMPIKT: a database of kinetic and thermodynamic mutant protein interactions. BMC Bioinformatics. 2018 Nov 27;19(1):455. doi: 10.1186/s12859-018-2493-7 . 30482172 PMC6260753

[30] Chen Peng, Li Jinyan, Wong Limsoon, Huang Jianhua, and Gao Xin, Accurate prediction of hot spot residues through physicochemical characteristics of amino acid sequences. Proteins, 2013, 81: 1351–1362. doi: 10.1002/prot.24278 23504705

[31] Chawla N V, Bowyer K W, Hall L O, et al. SMOTE: Synthetic Minority Over-sampling Technique. Journal of Artificial Intelligence Research, 2002, 16(1):321–357.

[32] Finn R D, Alex B, Jody C, et al. Pfam: the protein families database. Nucleic Acids Research, 2014, 42(D1):D222–D230. doi: 10.1093/nar/gkt1223 24288371 PMC3965110

[33] Attaf D, Djerriri K, Cheriguene R S, et al. One-Dimensional Convolution Neural Networks for Object-Based Feature Selection. Proc. SPIE 10789, Image and Signal Processing for Remote Sensing XXIV, 107891N (9 October 2018); doi: 10.1117/12.2325640

[34] Zhen C, Liu X, Li F, et al. Large-scale comparative assessment of computational predictors for lysine post-translational modification sites. Brief Bioinform. 2019 Nov 27;20(6):2267–2290. doi: 10.1093/bib/bby089 . 30285084 PMC6954452

[35] Bradley Andrew P. The use of the area under the ROC curve in the evaluation of machine learning algorithms. Pattern Recognition, 1997, 30 (7):1145–1159.

[36] Hall Mark, Frank Eibe, Holmes Geoffrey, Pfahringer Bernhard, Reutemann Peter, Ian H., The WEKA data mining software: an update. ACM SIGKDD Explorations Newsletter 2009, 11(1): 10–18.

[37] Liu Q, Chen P, Wang B, et al. Hot spot prediction in protein-protein interactions by an ensemble system. BMC Systems Biology, 2018, 12(S9). doi: 10.1186/s12918-018-0665-8 30598091 PMC6311905

[38] Suzek B E, Huang H Mcgarvey P, et al. UniRef: Comprehensive and Non-Redundant UniProt Reference Clusters. Bioinformatics, 2007, 23(10):1282–1288. doi: 10.1093/bioinformatics/btm098 17379688

[39] Rao R, Bhattacharya N, Thomas N, Duan Y, Chen X, Canny J, et al. Evaluating Protein Transfer Learning with TAPE. Adv Neural Inf Process Syst. 2019 Dec;32:9689–9701. . 33390682 PMC7774645

