# Supplementary material for: Prediction of hot spots towards drug discovery by protein sequence embedding with 1D convolutional neural network
Source: PLoS One. 2023 Sep 18;18(9):e0290899. doi: 10.1371/journal.pone.0290899 (PMC10506709; doi:10.1371/journal.pone.0290899)
Supplement: S1 File — (DOCX) [file pone.0290899.s001.docx]

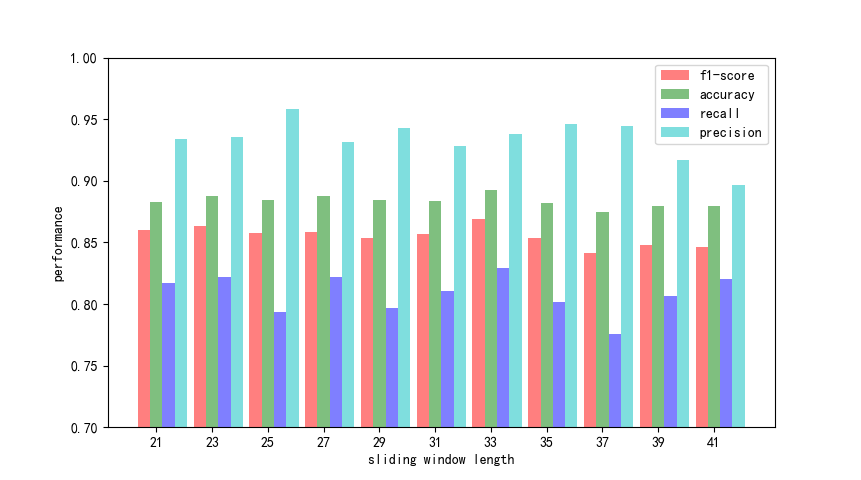


(a) Embed-1dCNN (SMOTE)


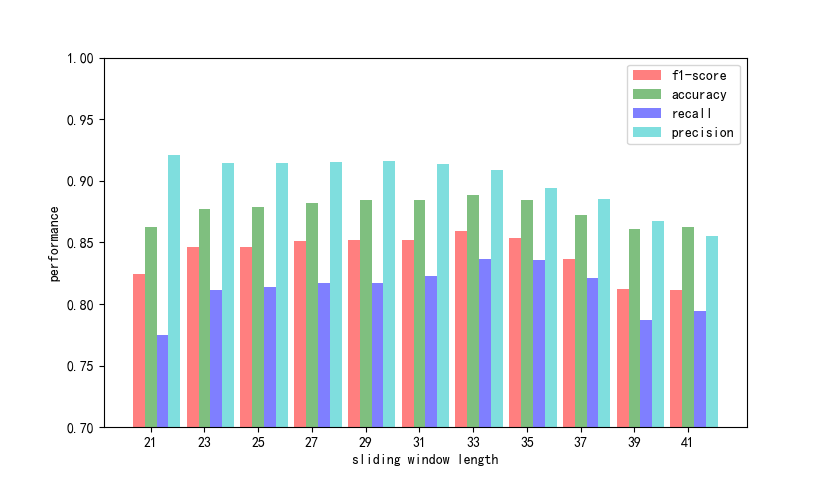


(b) Embed-1dCNN (class_weight)

**Figure 1.** The prediction results of the two groups of models on the four evaluation indicators
