# Supplementary material for: Prediction of hot spots towards drug discovery by protein sequence embedding with 1D convolutional neural network
Source: PLoS One. 2023 Sep 18;18(9):e0290899. doi: 10.1371/journal.pone.0290899 (PMC10506709; doi:10.1371/journal.pone.0290899)
Supplement: S2 File — (DOCX) [file pone.0290899.s002.docx]

| Model | Acc | F1 | Pre | Recall | Spe |
| --- | --- | --- | --- | --- | --- |
| Embed4117 | 0.8929 | 0.8692 | 0.9378 | 0.8289 | 0.9397 |
| Babbler-1900 | 0.8400 | 0.8100 | 0.8784 | 0.7736 | 0.9065 |
| Bert_base | 0.8379 | 0.8050 | 0.8909 | 0.7557 | 0.9029 |
| seqvec | 0.8446 | 0.8032 | 0.9270 | 0.7244 | 0.9379 |
| One-hot | 0.8286 | 0.7774 | 0.9222 | 0.7117 | 0.8947 |
| Embed100 | 0.7973 | 0.7559 | 0.8244 | 0.7344 | 0.8258 |

**Table 1.** Prediction results of six groups of protein sequence embedding models.
