# Supplementary material for: Prediction of hot spots towards drug discovery by protein sequence embedding with 1D convolutional neural network
Source: PLoS One. 2023 Sep 18;18(9):e0290899. doi: 10.1371/journal.pone.0290899 (PMC10506709; doi:10.1371/journal.pone.0290899)
Supplement: S3 File — (DOCX) [file pone.0290899.s003.docx]

**Suplementary File S3: Embedding models**

**1. Embed100**

In fact, Tristan Bepler and Bonnie Berger also added an encoder structure to the Embed4117. When it outputs a 4117-dimensional feature vector, it will be put into this encoder which includes a 3-layer BiLSTM structure and a linear layer and outputs 100-dimensional features [23]. The structure of Embed100 is shown in Figure 1.


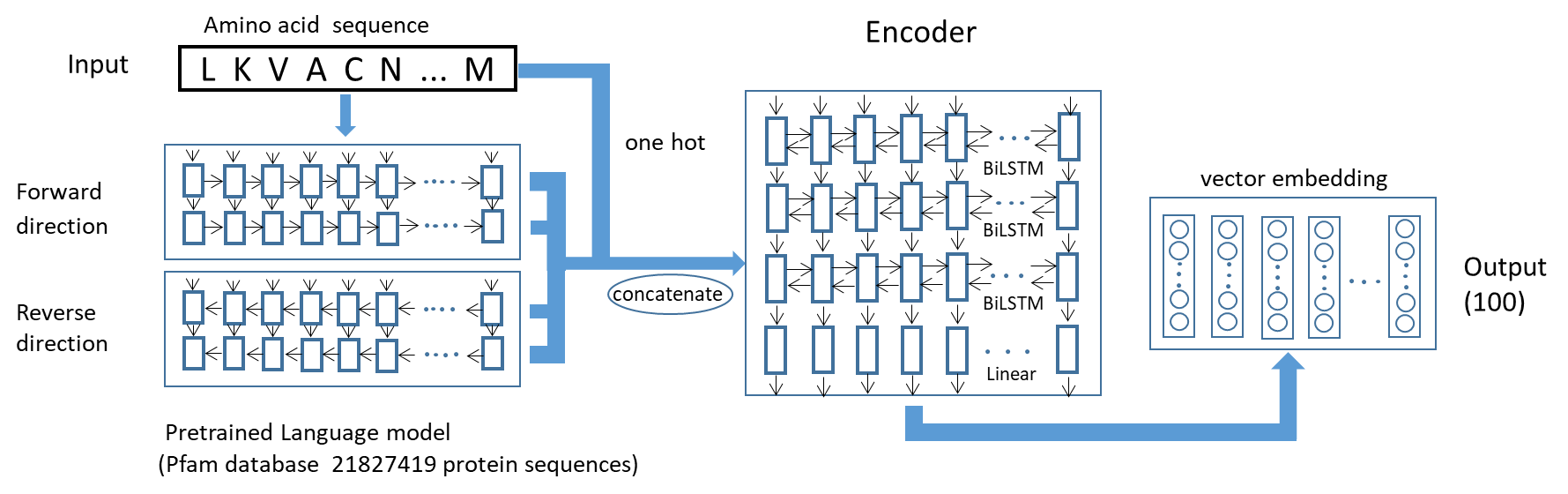


**Figure 1.** Model of Embed100

**2. seqvec**

Heinzinger *et al*. applied the NLP model ELMo to solve protein sequence related problems through transfer learning and trained the protein embedding model, called ‘seqvec’. The authors applied ‘seqvec’ to predict secondary structures at amino acid residue level, and predict the subcellular locations of ten types of proteins at protein level. As shown in Figure 2, ‘seqvec’ is composed of a three-layer network architecture. The first layer is a character-based CNN structure. Its function is to transform each amino acid residue into a 500-dimensional feature vector. This feature representation has no contextual information and is only related to the type of amino acid. The second and third layers are both composed of 512 bidirectional LSTM units. Their function is to combine amino acid residue categories with sequence information, so that the features of their context information of surrounding residues can be extracted. Finally, the model outputs a 1024-dimensional feature vector for each amino acid residue in protein sequence.


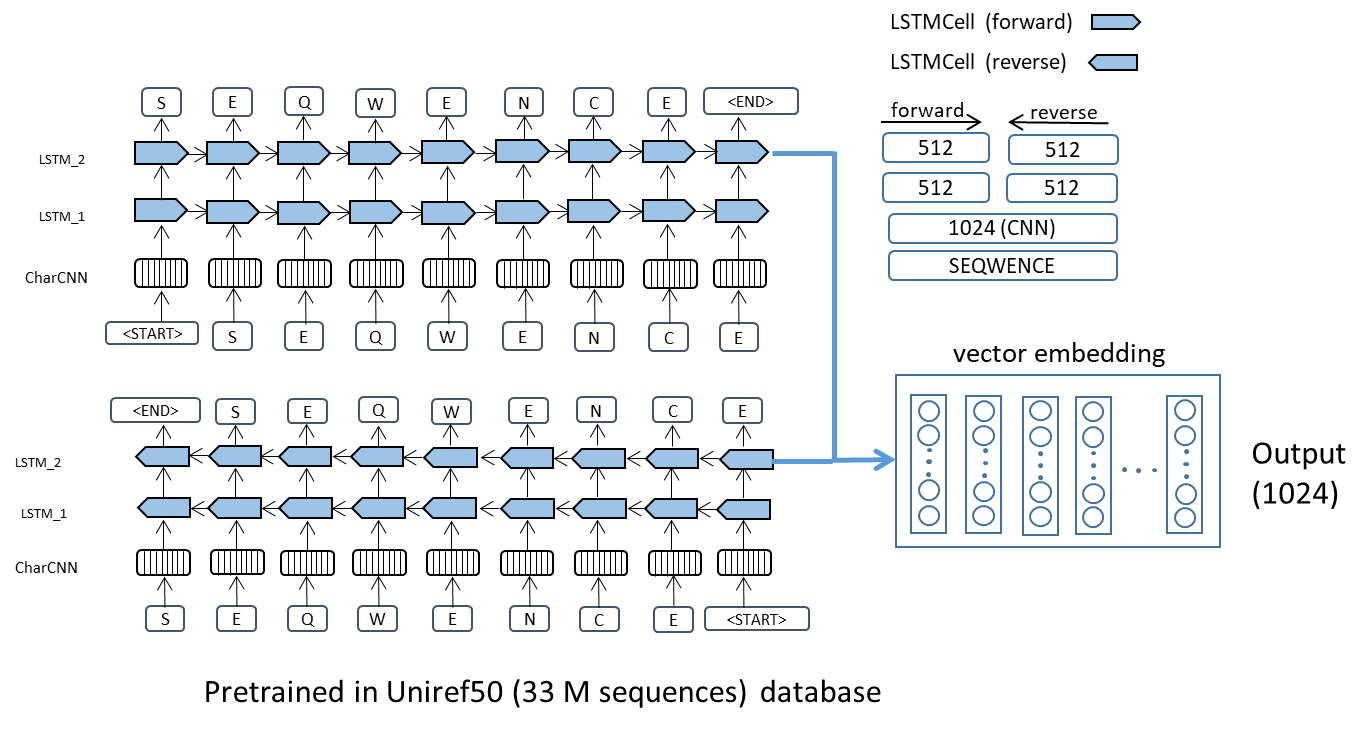


**Figure 2.** Model of seqvec

**3. Babbler-1900 and Bert_base**

Babbler-1900 embedding model was originally created by Ethan C. He trained a 1900-hidden unit multiplicative Long-Short-Term-Memory (mLSTM) Recurrent Neural Networks (RNNs) of amino acid character in UniRef50 [34]. The pretrained model of Babbler-1900 from TAPE was used in this work [35]. Bert_base is another pre-trained protein sequence embedding model in TAPE which is also a Multi-layer bidirectional transformer encoder. It outputs 768-dimensional features to represent each protein amino acid residue.

**4. One-hot encoding**

One-hot encoding schema is a common encoding method in the process of data preprocessing. The processed data is usually sparsely distributed in space. In this work, amino acids in one protein are represented in the form of 21 dimensions (the 21st dimension represents all other types of amino acid residues except the common 20 amino acids).
