# Supplementary material for: Prediction of hot spots towards drug discovery by protein sequence embedding with 1D convolutional neural network
Source: PLoS One. 2023 Sep 18;18(9):e0290899. doi: 10.1371/journal.pone.0290899 (PMC10506709; doi:10.1371/journal.pone.0290899)
Supplement: S4 File — (DOCX) [file pone.0290899.s004.docx]

**Suplementary File S4:** Setting of hyper-parameters for the proposed method


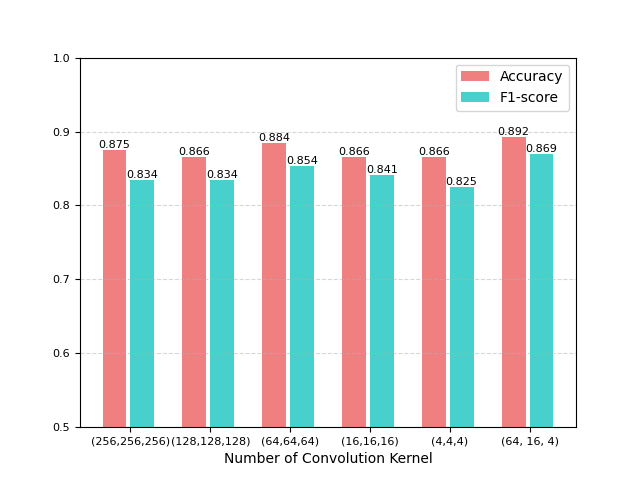


**Figure 1**. Performance comparison of the number of convolution kernels for the proposed method


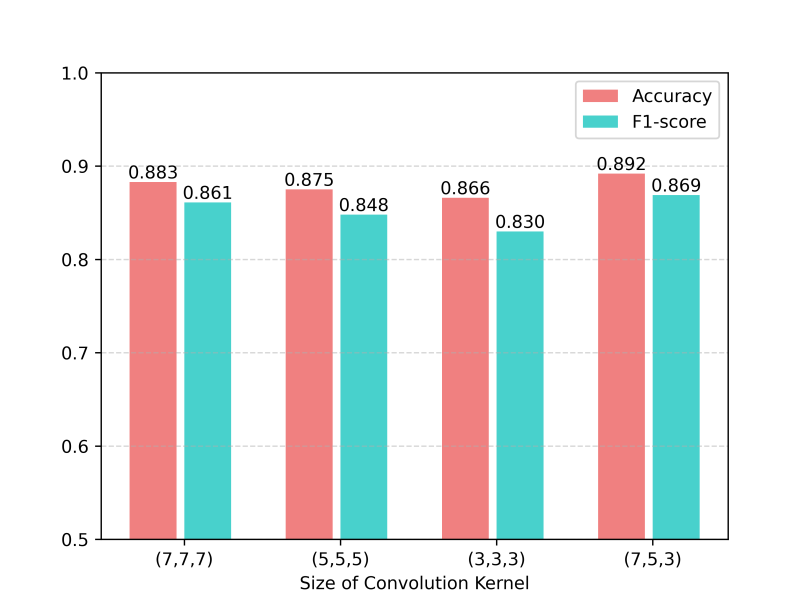


**Figure 2**. Performance comparison of size of convolution kernels for the proposed method


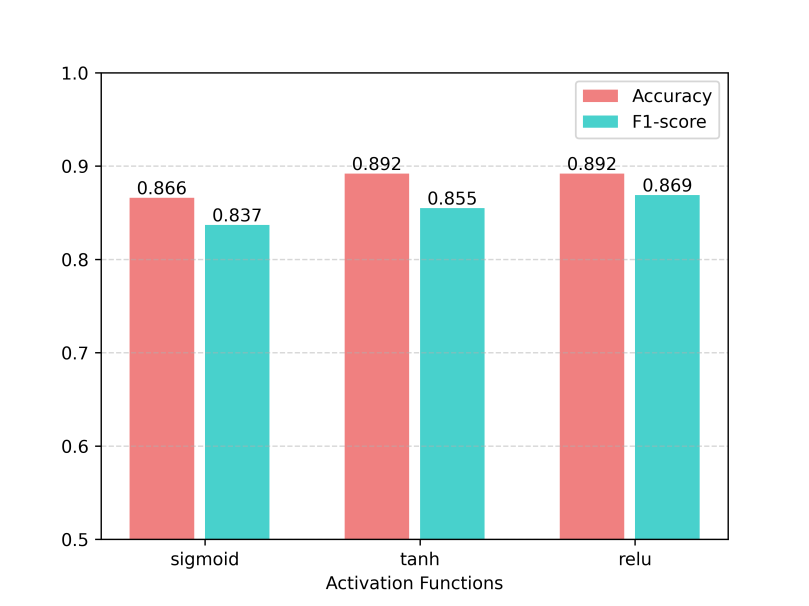


**Figure 3**. Performance comparison of the activation function for the proposed method
